# A Transparency Checklist for Carbon Footprint Calculations Applied within a Systematic Review of Virtual Care Interventions

**DOI:** 10.3390/ijerph19127474

**Published:** 2022-06-18

**Authors:** Oliver Lange, Julian Plath, Timo F. Dziggel, David F. Karpa, Mattis Keil, Tom Becker, Wolf H. Rogowski

**Affiliations:** 1 Department of Health Care Management, Institute of Public Health and Nursing Research, Health Sciences, University of Bremen, 28359 Bremen, Germany; keil@ipp.uni-bremen.de (M.K.); rogowski@uni-bremen.de (W.H.R.); 2 Leibniz ScienceCampus Digital Public Health Bremen, 28359 Bremen, Germany; 3 Professional Public Decision Making, Faculty of Cultural Studies, University of Bremen, 28359 Bremen, Germany; jplath@uni-bremen.de (J.P.); dziggeti@uni-bremen.de (T.F.D.); tomrunge@uni-bremen.de (T.B.); 4 Faculty of Business Studies and Economics, University of Bremen, 28359 Bremen, Germany; dkarpa@uni-bremen.de

**Keywords:** carbon footprint, carbon dioxide equivalents, greenhouse gas, digital health, virtual care, telemedicine, telehealth, e-health, digital public health, systematic review

## Abstract

Increasing concerns about climate change imply that decisions on the digitization of healthcare should consider evidence about its carbon footprint (CF). This study aims to develop a transparency catalogue for reporting CF calculations, to compare results, and to assess the transparency (reporting quality) of the current evidence of virtual care (VC) intervention. We developed a checklist of transparency criteria based on the consolidation of three established standards/norms for CF calculation. We conducted a systematic review of primary studies written in English or German on the CF of VC interventions to check applicability. Based on our checklist, we extracted methodological information. We compared the results and calculated a transparency score. The checklist comprises 22 items in the aim, scope, data and analysis categories. Twenty-three studies out of 1466 records were included, mostly addressing telemedicine. The mean transparency score was 38% (minimum 14%, maximum 68%). On average, 148 kg carbon dioxide equivalents per patient were saved. Digitization may have co-benefits, improving care and reducing the healthcare CF. However, the evidence for this is weak, and CF reports are heterogeneous. Our transparency checklist may serve as a reference for developing a standard to assess the CF of virtual and other healthcare and public health services.

## 1. Introduction

There is a growing awareness that global warming is a major threat to global health [1]. Given that public healthcare is a significant contributor to global greenhouse gas (GHG) emissions [2], there are various calls for action on climate change in the healthcare sector [3]. Identifying climate change mitigation potential in healthcare is particularly attractive because there may be substantial co-benefits for climate and public health action [4].

There is a need for comparative evidence to identify such potential. In recent decades, medicine and public health have experienced notable progress in evidence-based decision-making [5]. The scientific community has developed a variety of consented reporting standards to facilitate standardised evidence assessments (e.g., [6]). For example, the Consolidated Health Economic Evaluation Reporting Standards (CHEERS) [7] is aimed at improving the transparency of health economic evaluations which can be used for healthcare coverage decisions. Based on the idea of defining comparative methodological standards, we consider which elements of a carbon footprint calculation should be reported. This may increase the studies’ transparency and consistency, which is important for making well-informed decisions.

The GHG emissions of a particular good or service are generally assessed in terms of its carbon footprint (CF), the sum of GHG emissions and removals caused by a product or service, expressed as carbon dioxide equivalents (CO_2_e) [8]. Currently, there are three leading guidelines for assessing product or service CF. All of them assess carbon emissions along a product’s life cycle and are oriented to the life cycle assessment (LCA) standards ISO 14040 and ISO 14044, issued by the International Organization for Standardization (ISO) [9,10]. First, the ISO issued a standard that defines principles, requirements and guidelines for quantifying product CF. It was last updated in 2018 (ISO 14067:2018) [8]. This guideline forms part of the ISO 14060 norm family that also addresses the CF of organisations (ISO 14064-1) and projects (ISO 14064-2). The norm family sets out requirements for verifying GHG statements (ISO 14064-3) for bodies that validate and verify GHG statements (ISO 14065) and for competencies of validation and verification teams (ISO 14066). Second, there is the Greenhouse Gas Protocol Product Life Cycle Accounting and Reporting Standard (Product Standard) published by the World Resources Institute and the World Business Council for Sustainable Development [11]. Like ISO 14067, the GHG protocol also provides a complimentary standard for organisations (Corporate Standard) and projects (Project Standard). In addition, it provides standards for the mitigation goals of cities and for policy goals and actions and different types of guidance. Third, there is the Publicly Available Specification (PAS 2050:2011) [12] published by the British Standards Institution (BSI), Department of Environment Food and Rural Affairs and the Carbon Trust. PAS 2050 was the first of the three guidelines to be published (first published in 2008 and revised in 2011).

Generally, the standards are largely compatible. However, they differ in structure, wording and detail: ISO 14067 is written in technical language, with multiple cross-references to other paragraphs within the same or other ISO standards. The Product Standard is less technical and provides direct requirements and guidance for each LCA inventory step. The Product Standard and the ISO 14067 contain a section specifically dedicated to reporting. PAS 2050 follows a different structure and is much shorter and less detailed regarding specific reporting. Initially, to our knowledge, there was no standardised transparency catalogue synthesising the three guidelines that was suitable for use in assessing reporting quality in systematic reviews of CFs. Hence, our first aim was to develop a transparency catalogue. During this period of development, two systematic reviews were published, each developing a tool for assessing quality/transparency [13,14]. Although Rizan et al. [13] refer to the same three guidelines, their catalogue appears to be based primarily on the GHG protocol [11] rather than on a transparent synthesis of the different guidelines. The catalogue of Drew et al. [14] is not based on specific CF guidelines but rather on guidelines for critically reviewing LCA [15].

One recent trend in medicine and public health for which evidence about CF would be desirable is digitization, for example the increasing use of information and communication technologies (ICT) in health care and public health. Digital health is an important item on the agenda of healthcare policymakers [16]. However, digital health includes a wide range of interventions. One established concept is ‘mobile health’ [17,18]. This refers to the connection of healthcare stakeholders over a distance in real time using communication technology to perform health and healthcare services [19]. This is also the definition of ‘virtual care’ (VC) used by the World Economic Forum [19]. VC can be subdivided into telemedicine, which specifically refers to clinical healthcare services, and telehealth, which encompasses broader services such as fitness tracking or nutrition advice. To focus on a homogenous group of digital interventions, we investigate the CF of VC in the following.

Digital technologies have the potential to reduce the CF of healthcare. For example, they can replace the personal transport and commuting of patients, increase logistics efficiency, and optimise the energy consumption of healthcare facilities. However, the digital economy features a large and increasing CF because the increasing energy efficiency of digital devices is more than offset by the growing number, power, complexity and range of applications of these new devices [20]. Furthermore, it stimulates demand for energy-intensive infrastructures like 4G networks [21]. In addition, apart from the GHG emissions of ICT alone, the embedded emissions of intermediate inputs from non-ICT sectors like electricity and basic materials also need to be considered. These indirect impacts are many times greater than the direct ones [22].

A recent review showed that the CF of digitization strategies varies depending on methodological assumptions made in CF analyses (e.g., about boundaries and thus the amount of embedded GHG emissions included), and typically it overestimates energy savings [21]. Therefore, methodological rigor and reporting transparency are crucial in assessing the CF of digital health. Purohit et al. [23] compare the results of the CF analysis of telemedicine. They focus on quantitative results and do not assess the transparency and methodological details of the featured studies.

This study aims to develop a transparency catalogue for reporting CF calculations in the health context, compare the results of CF calculations, and assess the transparency (reporting quality) of the current evidence of digital health interventions focusing on VC.

## 2. Methods

### 2.1. Development of a Transparency Catalogue

We selected the methodological items that appear in all three standards to develop a consolidated transparency catalogue. We chose ISO 14067 as a starting point because it is the most detailed standard. From section seven (CF study report) in ISO 14067, we extracted the mandatory items to be reported in CF analyses. In the second step, we assessed which of these items also appeared in the requirements set out by the GHG Protocol Product Standard [11]. In the third step, we assessed which of the items contained in a CF assessment report according to ISO 14067 and GHG Protocol Product Standard are consistent with the requirements set out by PAS 2050 [11]. Given that the structure and wording of the standards differ, this process was not strictly deductive. If an item was prominent in the Product Standard but not identified in ISO 14067, we conducted a second search for this item in the ISO standard. The items sometimes needed to be split or merged. Two of the authors (WR and JP) independently continued this iterative process until they had identified an agreed upon set of required items. Subsequently, differences were resolved by consensus to arrive at a final list of items for use in this study.

We described each item based on information provided in the guidelines and formulated a question for each one, answerable by yes or no responses. This process resulted in a catalogue for assessing methodological transparency. If methodological choice allowed for more than one response, we split the items into separate, mutually exclusive sub-items to ensure that the final number of reported items can always be divided by the same denominator. For example, the guidelines may require that CF be reported in CO_2_e, which includes other GHGs. However, if carbon dioxide (CO_2_) is the only relevant GHG, it may be acceptable to omit ‘equivalents’. A corresponding item asked: (a) Is CF reported in CO_2_e?; or (b) If CF is reported in CO_2_ only, is it justified that this is the only relevant GHG? We then ordered the items and assigned them to categories for convenient use. Convenience meant that, by extracting information on the items, reviewers obtained not only an overview of methodological transparency but also a legible overview of the content of the studies. Using the first half of the finally included studies, we piloted and adjusted the formulation, categorisation and ordering of items in the transparency catalogue iteratively until the catalogue seemed operational.

### 2.2. Search Strategy and Selection Criteria

We conducted a systematic review for CF analyses of digital health interventions focused on VC, oriented to PRISMA 2020 guidelines [24,25]. In explorative searches, we identified studies combining the concepts of CF assessment and digital/VC interventions. The search strategy consisted of common keywords and theoretically deduced keywords (see Supplementary S1). We searched the PubMed, Web of Science, Scopus, CINAHL and EconBiz databases on 22 November 2019.

We screened titles and abstracts to select studies for full-text investigation if they met the following criteria: (1) the study provided a calculation of GHG emissions, (2) the object of investigation was a VC service, (3) the study was a primary study and (4) the language of the abstract was English or German. The full-text investigations were based on the same selection criteria, with the addition to criterion (1) that the study had to provide at least some information about the CF calculation method. Pairs of two independent reviewers (T.F.D. and T.B. or D.F.K. and J.P.) did the title-abstract screening, and disagreements were resolved by a third independent reviewer (T.F.D. or D.F.K.). Two reviewers (J.P., D.F.K. or W.H.R.) independently checked full-text eligibility for the inclusion of studies.

We screened titles and abstracts based on PubMed search alerts up to 3 January 2022 in order to update our systematic search. Two independent reviewers screened titles and abstracts and investigated full texts (M.K. and O.L.). Disagreements were solved by consensus.

### 2.3. Data Extraction and Analysis

To assess whether an item was reported, we searched the studies for the methodological information to which the item was related. If identified, we extracted this information; otherwise, we documented ‘not reported’. W.H.R. and J.P. extracted the data independently and resolved disagreements by consensus. To provide a structured overview of the study results, we inductively developed categories for the types of information provided and calculated the number of times each type of information was reported for each transparency catalogue item. M.K. and J.P. extracted information independently in the search update, while O.L. solved disagreements. M.K. categorised the extracted items for the graph where applicable.

We calculated a score, dividing the number of reported items by the number of items contained in the transparency catalogue to provide a quantitative indicator of reporting transparency. Where applicable, for each assessed intervention, O.L. and F.W. independently extracted the total savings in CO_2_e or CO_2_ per patient or per consultation to obtain a comparable estimate of the impact of VC on GHG emissions. We calculated the savings in CO_2_e per patient, CO_2_e per consultation, CO_2_ per patient and CO_2_ per consultation by dividing total CO_2_e or total CO_2_ and patient or consultations. If a study had savings per patient or per consultation, we extracted this value directly.

## 3. Results

We report the results in two sections. First, we present the results of the development of our transparency catalogue, and second, we report the systematic review results.

### 3.1. Development of Transparency Catalogue

**Figure 1:**
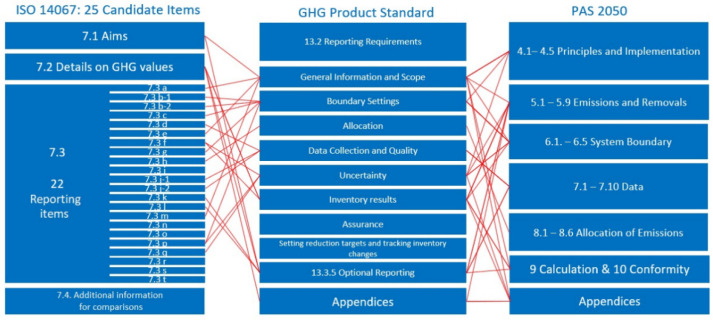
Consolidation of transparency catalogue.

Figure 1 shows the consolidation process of items developed from ISO 14067 with GHG protocol and PAS 2050.

The three guidelines were largely overlapping and consistent regarding the general approach to CF analysis and major topics. However, when looking at single items, some differences appeared. In section seven of ISO 14067, we identified 22 separate items that should generally be reported and eight items to be reported only if applicable. In addition, we identified two items that were implied by the general prescriptions of the norm but which were not formulated as reporting items. Of these 32 items, seven were not identified as explicit items in the GHG Protocol Product Standard, and two were optional only. The remaining 23 items contained three that were optional only in ISO 14067 and excluded or optional in PAS. Therefore, we identified 20 items that were included in at least two standards and were not in contradiction with the third. We rearranged these 20 overlapping items to the 22 items described in Table 1. Further descriptions of the items can be found in Supplementary S2.

### 3.2. Results of the Systematic Review

**Figure 2:**
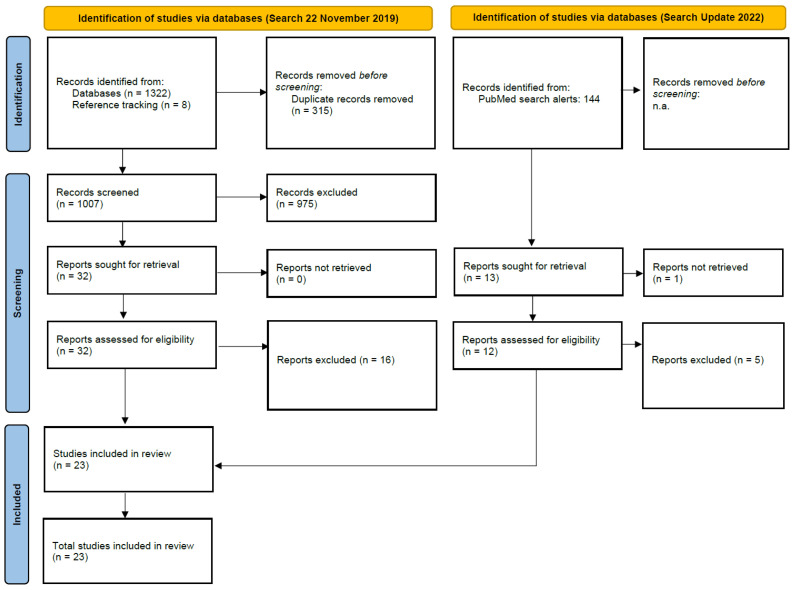
Result of review process based on (modified) PRISMA flow chart [24].

The main systematic search identified 1332 records. After removing duplicates, we screened 1007 titles and abstracts. Thirty-two studies were identified for full-text investigation. Sixteen articles were excluded because they had no primary study, contained no CF calculation, or had no VC topic. Finally, 16 studies were included in the systematic review [26,27,28,29,30,31,32,33,34,35,36,37,38,39,40,41]. The search update includes 144 titles and abstracts. After investigating 13 full texts, seven additional studies were included [42,43,44,45,46,47,48]. Finally, 23 studies were included. Figure 2 depicts the identification process based on the PRISMA 2020 Flow Chart [24].

Most of the studies assessed telemedicine, such as, for example, the delivery of health services via remote telecommunications. The specific type of services provided by telemedicine services was not always clear and likely varied. One study addressed prevention (support of smoking cessation). None of the studies addressed primary care (i.e., care by providers first seen by patients, such as general practitioners). Most frequently, these involved secondary care (i.e., more intensive treatment like hospital care for acute conditions) or tertiary care (i.e., specialized consultative health care like oncologists). One study addressed quaternary care (i.e., care which is specialized at a very high level which may include experimental care, in this case treatment in an academic hospital), and one study addressed a support system (the use of electronic health records).

If the studies reported a functional unit, it was mostly one treated patient. Information about the reference flow of resources needed to perform the functional unit was sparse. Although most studies considered the kilometres saved by a medical institution, only a few studies included emissions due to electricity consumption by technical equipment or data transmission. Hardly any of the studies involved a life cycle perspective and included the resource flows prior to and after performing the digital health service itself. Similarly, in the CF analyses, we rarely identified lists of unit processes, exclusions and reasons for exclusions or precise information about system boundaries.

The studies relied on different types of primary and secondary data. Typically, they used public sources for emission factors, for example, the average GHG emissions from automotive travel in different countries. In combination with these data, the authors of studies frequently used patient addresses stored in secondary institutional data to estimate travel distances (avoided). Some of the studies also used data from patient surveys. None of the studies assessed the temporal representativeness of the data. Most frequently, the authors accounted for geographical representativeness only by using national data on travel emissions. Only in a few cases did the authors account for additional aspects, such as the emission factors of regional energy grids. They also typically did not report on technological representativeness, and none of the studies addressed the completeness of data.

**Figure 3:**
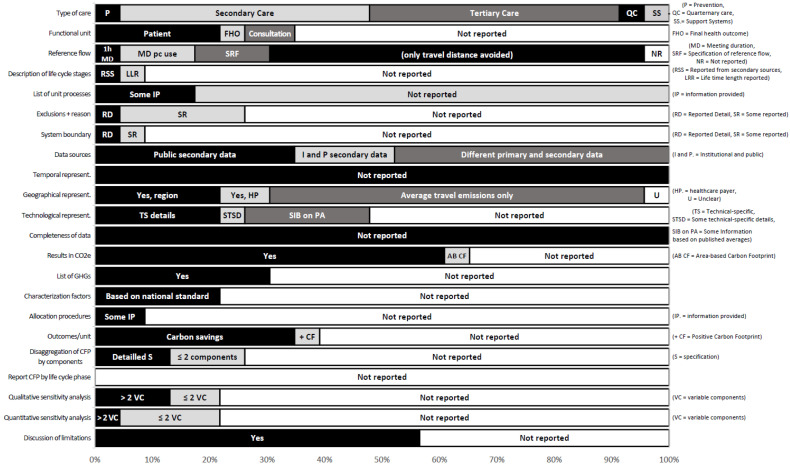
Overview of study results reported by item.

The studies rarely reported outcomes in terms of CO_2_e per unit of analysis. If they did so, they mostly reported the carbon savings. In limited instances, they reported CF by specific component and never by life cycle stage. Even if most studies reported some limitations of their studies, we typically could not identify quantitative or qualitative sensitivity analysis. Figure 3 provides an overview of the study results by reporting item. Supplementary S3 provides detailed study information on the extracted items.

On average, 31% of the items from our transparency catalogue were reported in the investigated studies. The maximum number of reported items is 64%. The minimum number of reported items is 9%.

Regarding the results, 11 studies reported savings expressed in CO_2_ equivalents per patient or the data necessary for the calculation. The average amount saved was 148 kg CO_2_e per patient. Six other studies reported CO_2_e savings per consultation, with an average of 128 kg CO_2_e per consultation. In some cases, it was not clearly defined whether one patient corresponded to one consultation. Several studies did not report results in CO_2_e but in CO_2_ (and other GHGs). Therefore, the average CO_2_ savings per patient (respectively per consultation) was 109 kg CO_2_ (respectively 31 kg CO_2_). It must be stated that the averages are based on different studies, depending on whether the respective values were reported. Two studies did not provide savings but only a total for the CF. One study provided an ecological footprint instead of a CF. Details of the results and calculation of averages are provided in Supplementary S4.

## 4. Discussion

### 4.1. Statement of Principal Findings

Mitigating climate change is an important policy objective that applies to healthcare and public health. Just as individual care and public coverage decisions should be informed by the best available evidence of effectiveness, there is a need for evidence about CF to support decision-makers in promoting more climate-friendly health and health care. Digital health is an example of the complexity of resource flows that should be considered in calculating CF. In addition to the direct effects, namely the GHG emissions of energy consumed by digital information transfer and of unnecessary travel avoided by telemedicine, GHG emissions associated with devices and network infrastructure may also need to be reported. There is a need for consented assessment standards to ensure that the authors of CF analyses account for and report such methodological considerations.

In general, we found that the existing studies mainly addressed telemedicine and typically reported that telemedicine is associated with carbon savings. However, the studies frequently ignored several standard methodological requirements of CF analyses. In particular, they typically did not include a life cycle perspective and did not report carbon emissions embedded in devices and the technical infrastructure. For the most part, they did not report on the important question of the system boundaries and associated methodological questions about exclusions of unit processes and reasons for exclusion. Furthermore, they only addressed methodological items like the completeness or the representativeness of data for the system under investigation to a very limited extent.

To our knowledge, this is the first study that analysed the joint requirements of the three major standards of CF analysis to facilitate evidence-based reviews of CF studies in healthcare. Independent of this study, two other catalogues for appraising the transparency of CF analyses were developed. Rizan et al. [13] analysed the CF of surgical operations. Based on the GHG protocol, they extracted endpoints. While extraction and quality assessment are based on the GHG protocol only, it could not be identified if any other guidelines had an influence on the extraction of endpoints. Extraction and quality assessment include items of completeness, consistency, transparency and accuracy. Although the goal of assessing the reporting quality of CF calculations is the same, the process of how items from PAS and ISO are included or excluded is not presented in a fully comprehensible way. The comparison of CF results is not possible because surgical operations and VC are different topics.

Drew et al. [14] investigated life cycle assessments of surgical and anaesthetic care. They conducted a critical appraisal based on Weidema [15] and included items of internal validity, external validity, consistency, transparency and bias [14]. Their appraisal criteria are more extensive, involving 35 (sub-)items in total, structured by the four phases of goal and scope (13 items), inventory analysis (seven items), impact assessment (six items), and interpretation (nine items). However, they focus on life cycle assessments. Therefore, they use the generic ISO standard for life cycle assessments, in contrast to our study that used different consolidated standards for CF calculation. Although there are many commonalities, there are also some differences. Our checklist requires a list of unit processes, while Drew et al. [14] ask for explicit data collection more generically; our checklist explicitly asks for temporal, technological and geographical representativeness of the data, while Drew et al. [14] ask for representativeness generically; only our checklist explicitly assesses whether the completeness of data was assessed; our checklist explicitly asks whether CF was expressed in CO_2_ or CO_2_e, while Drew et al. [14] ask for impact categories generically; our checklist asks explicitly for a list of GHGs included (or, if only CO_2_ was included, a justification that this is the only relevant GHG); this checklist asks for GHG characterisation factors while Drew et al. [14] ask for the characterisation method generically; only our checklist asks for CF by component (biogenic vs non-biogenic).

In contrast to the mentioned transparency catalogues, Purohit et al. [23] focused on comparing results based on the medium used for telehealth consultations, i.e., synchronous telephone consultation, synchronous video consultation and non-synchronous consultation [23]. They concluded that telephone consultation saves more emissions than video consultation. While they report carbon savings between 0.70 to 372 kg CO_2_e per consultation, our reviews show savings of CO_2_e per consultation of 109 kg (Min: 285; Max: 0.17 kg). However, the proportion of studies that allow CO_2_e per patient as an outcome is higher in our review. In the discussion section, the authors mentioned methodological limitations within the included studies. However, they did not compare methodological choices or assumptions within the studies and did not conduct a quality assessment of the included studies.

### 4.2. Limitations

There are some methodological limitations concerning the development of the transparency catalogue, particularly concerning the consolidation of requirements by different standards. First, determining reporting items was not always possible in an unambiguous manner. This is because reporting requirements can be presented in terms of how something needs to be reported or in terms of what needs to be reported. Certain types of information can be implied or they can be explicitly stated. For example, "goal and scope definition" is a central item in the Product Standard. ISO 14067 only states that type and format shall be defined in the goal and scope definition phase of a study so that it appears as an item external (prior) to the report itself. In this instance, interpretations were required; for example, we assumed that it is an implied item that should be reported.

Second, we did not account for all standards in an equal manner. In assessing PAS 2050, we did not restrict the list of items to those that are required explicitly but rather assessed whether the items identified based on ISO 14067 and the Product Standard are consistent with the broad requirements of PAS 2050. We assumed that this is consistent with PAS 2050 because the latter concerns the recording rather than the public reporting of CF. Furthermore, we excluded additional standards from our analysis, such as the Climate Declaration, BP X30-323, or the Japanese CFP Communication Programme. This seemed appropriate because ISO 14067, Product Standard and PAS 2050 appear to be the leading standards.

Third, this study did not attempt to harmonise standards of how to calculate CF, only providing an agreed minimum standard of what should be reported in studies calculating CF. It does not provide sector-specific guidance for calculating CF in (digital) health and healthcare. This was beyond the scope of our study. Therefore, further work would be necessary to develop sector-specific guidance for estimating the CF of health and health care interventions. Fourth, this study provides a tool to create evidence about the methodological transparency of the studies, not to rate their quality. Therefore, our focus is mainly on the methods used and not the quality of outcomes. We also focus only on climate change as one of the nine planetary boundaries [49], thereby limiting the assessment to one environmental problem while omitting measures to address the others (see also ISO 14067 Appendix A on limitations of CF).

There were also some limitations regarding the systematic review we conducted. We only included studies written in German and English. This review only included VC interventions. This restriction was made for better comparability of a common group of studies. Digitization plays an increasingly important role in healthcare and public health, so it would also be appropriate to apply the topic to other digital (public) health areas. Although we conducted all steps of the systematic search with two independent pairs of researchers (especially data extraction), the summary of the results in the central result graphic was done by a single person. For this reason, we added the original consolidated extracted information as a Supplementary Materials. Moreover, we included a variety of primary studies in our review. Therefore, we comprehensively examined all possible evidence and types of calculations. However, we included calculations with a lower level of evidence. In some cases, our transparency catalogue consists of items labelled (a) and (b); for reasons of initial piloting of this checklist, we summarised these as single items in the transparency score.

There are different initiatives for improving the evidence basis of CF analyses. The three standards, specifically the Product Standard, play a major role in this process. The transparency catalogue developed in this study serves the complementary aim of providing a cross-standard assessment tool. Such a tool faces the limitation that it is less precise than any individual standard.

### 4.3. Implications for Practice and Further Research

Given that currently there are at least three different checklists for assessing CF studies, it appears timely to validate the catalogue of items presented here in a transparent, participative process of formulating reporting requirements similar to other EQUATOR guidelines. Considering that CF analysis in healthcare is still an emerging field, we hope that the transparency catalogue presented here can serve as the first step in preparing such a process. Furthermore, although sector-specific guidance would be valuable for CF analysis in healthcare, developing such guidance would require collaboration among key stakeholders [11]. Even if this checklist is oriented to the requirements of healthcare, it cannot provide such guidance, but may provide input to such a process of development.

Future CF calculations may be more detailed based on the developed checklist. In terms of digital health interventions, it could be a particular consideration of allocation aspects. Therefore, the CF of trips to an urban clinic may be combined with private undertakings (e.g., shopping), or devices may be shared for other non-health purposes. Other aspects are a clear definition of a functional unit for better comparability with other CF calculations.

For evidence-based decision practice, critically assessing the evidence is essential. This includes evaluating the reporting quality against a benchmark of elements that are to be included into a CF calculation. The transparency catalogue developed in this study can help decision-makers by providing such a benchmark. Furthermore, this systematic review of VC interventions shows that this type of digital health application can be carbon-saving. In the future, implementing new health services (not only in the field of digital health) can include not only aspects of cost-effectiveness but also environmental impacts.

Evidence-based decision practice requires evidence standards. By now, standards mainly address effectiveness and cost-effectiveness. The checklist provided here may enhance the remit of evidence-based practice also to include the new field of CF, which is likely to increase in importance in healthcare, public health and beyond.

## 5. Conclusions

There is a need for evidence-based guidance on the climate impact of different types of healthcare and public health interventions. This study provides a benchmark for assessing the quality of evidence of CF analyses in healthcare explicitly based on the three major guidelines for CF. Further work is needed to build on the work in this study and in other reviews to develop consented reporting requirements within a transparent, participative process similar to established guidelines (see the EQUATOR network).

While CF is an attribute of new (digital) healthcare and public health technologies, this study demonstrated that the evidence in this field is still weak. In addition to the need for agreed upon guidelines, there is a need for more studies on this important topic to facilitate an evidence-based transition to climate-friendly (digital) healthcare and public health services.

## Figures and Tables

**Table 1:** Methodological items of carbon footprint analyses.

	Item	Assessment Question	How to Extract
Aim	1	Does the study specify its aim, e.g., in terms of the product or service for which CF is assessed?	Assess whether the healthcare goods or services subject to the study are described; if yes, extract a brief description. In particular, extract the name of the product subject to the assessment; its comparator and the aim of comparison if applicable; and its predecessor and the aim of performance tracking if applicable.
	2a	Does the study specify the functional unit?	Assess whether a functional unit is specified; if yes, extract the functional unit.
	2b		Assess whether no final use of the product is known and/or whether the study explicitly justifies the limitation of a partial carbon footprint; if yes, report ‘Partial CF’.
	3	Does the study specify the reference flow?	Assess whether the reference flow is specified; if yes, extract information about the reference flow.
	4	Does the study provide a description of the life cycle stages?	Assess whether the life cycle phases of the product or service under investigation are explicitly addressed and described; if yes, extract stated life cycle phases.
	5	Does the study provide a list of important unit processes?	Assess whether a list of unit processes is provided; if yes, extract the list of unit processes.
	6	Does the study specify exclusions and reasons for exclusions?	Assess whether exclusions of unit processes or single energy or material flows are reported; if yes, extract a list of data exclusions.
	7	Does the study specify the system boundary?	Assess whether, in the methods section, the system boundary is specified and justified. If yes, extract information on the system boundary.
Data	8	Does the study provide sources for all data used in the analysis?	Assess whether all data sources are provided (these may include primary and secondary data); if yes, extract all data sources.
	9	Does the study assess the temporal representativeness of the data?	Assess whether the data year or other details about the period for which the data are relevant are reported; if yes, extract exemplary information on temporal representativeness.
	10	Does the study assess the geographical representativeness of the data?	Assess whether information about the geographical region to which the data apply is reported; if yes, extract exemplary information on geographical representativeness.
	11	Does the study assess the technological representativeness of the data?	Assess whether information about the technology for which the data are relevant is reported; if yes, extract exemplary information on technology coverage.
	12	Does the study assess the completeness of the data?	Assess whether information about the completeness is provided; if yes, extract this information.
Analysis	13	Does the study estimate CF in terms of CO_2_e?	Assess whether an outcome is specified in terms of CO_2_e [Ref. ISO 14067:2018, 7.2]; if yes, state ‘yes’.
	14a	Does the study provide a list of GHGs taken into account?	Assess whether a list of GHGs taken into account is provided [Ref. ISO 14067:2018, 7.3 e)]; if yes, extract included GHGs.
	14b		If CO_2_ is analysed only, has it been justified as to why this is the only relevant GHG? If yes, extract the justification on which it was based (see also item 5)
	15a	Does the study specify the selected characterisation factors?	Assess whether characterisation factors are reported; if yes, extract the source of the values.
	15b		If CO_2_ is analysed only, has it been justified why this is the only relevant GHG? If yes, extract justification it was based on (see also items 5 and 12b).
	16a	Does the study report the selected allocation procedures?	Assess whether allocation procedures are addressed. If yes, extract shared processes and allocation procedures.
	16b		If no allocation is addressed, was it why allocation is not relevant to the study justified? If yes, extract justification (see also item 5).
Results	17	Does the study report the outcomes per unit of analysis?	Assess whether data on CF per unit of analysis is provided; if yes, extract the figure and the unit of analysis.
	18	Does the study report CF separately per specific component?	Assess whether CF is reported separately per component; if yes, extract component and CFP per component (which may include that some components of emissions or removals amount to zero).
	19	Does the study report CF according to life cycle phases?	Assess whether total GHG emissions are differentiated by life cycle phases; if yes, extract data.
	20	Does the study report a qualitative statement on the influence of key uncertainties or methodological choices on the result?	Assess whether the impact of at least one uncertainty or methodological assumption on results is reported; if yes, extract the most influential ones.
	21	Does the study perform a quantitative sensitivity analysis?	Assess whether quantitative sensitivity analyses are reported; if yes, extract type of sensitivity analysis (e.g., one-way or two-way sensitivity analysis, tornado diagram, probabilistic analysis).
	22	Does the study critically discuss limitations, e.g., appropriateness of system boundary, data quality, or methods of analysis?	Assess whether limitations of the CF study are critically discussed; if yes, extract exemplary reported limitations.

## Data Availability

Not applicable.

## Supplementary Materials

The following supporting information can be downloaded at: https://www.mdpi.com/article/10.3390/ijerph19127474/s1, Supplementary S1: Search strategy; Supplementary S2: Further description of transparency catalogue; Supplementary S3: Detailed information on the extracted study items; Supplementary S4: Quantitative results of the included carbon footprint calculations.
