# Supplementary material for: A Transparency Checklist for Carbon Footprint Calculations Applied within a Systematic Review of Virtual Care Interventions
Source: Int J Environ Res Public Health. 2022 Jun 18;19(12):7474. doi: 10.3390/ijerph19127474 (PMC9223517; doi:10.3390/ijerph19127474)
Supplement: Supplementary file 1 [file ijerph-19-07474-s001.zip › Supplementary S1_Search Strategy.pdf]

Supplementary S1: Details of search strategy

Database	Searchterm (Date of search 22.11.2019)	Hits
PubMed - Search Details	(("carbon footprint"[MeSH Terms] OR ("carbon"[All Fields] AND "footprint"[All Fields]) OR "carbon footprint"[All Fields]) OR footprint[All Fields] OR (ecological[All Fields] AND footprint[All Fields]) OR ("greenhouse gases"[MeSH Terms] OR ("greenhouse"[All Fields] AND "gases"[All Fields]) OR "greenhouse gases"[All Fields] OR ("greenhouse"[All Fields] AND "gas"[All Fields]) OR "greenhouse gas"[All Fields]) OR "life cycle assessment"[All Fields] OR co2eq[All Fields] OR co2e[All Fields] OR (("carbon dioxide"[MeSH Terms] OR ("carbon"[All Fields] AND "dioxide"[All Fields]) OR "carbon dioxide"[All Fields]) AND equivalent[All Fields]) OR (co2[All Fields] AND equivalent[All Fields]) OR co2-eq[All Fields] OR (("carbon"[MeSH Terms] OR "carbon"[All Fields]) AND emission[All Fields]) OR (("carbon"[MeSH Terms] OR "carbon"[All Fields]) AND reduction[All Fields])) AND (("telemedicine"[MeSH Terms] OR "telemedicine"[All Fields]) OR ("remote consultation"[MeSH Terms] OR ("remote"[All Fields] AND "consultation"[All Fields]) OR "remote consultation"[All Fields] OR "teleconsultation"[All Fields]) OR telecare[All Fields] OR telediagnosis[All Fields] OR ("telemedicine"[MeSH Terms] OR "telemedicine"[All Fields] OR "telehealth"[All Fields]) OR telemonitoring[All Fields] OR tele-medicine[All Fields] OR tele-consultation[All Fields] OR tele-care[All Fields] OR tele-diagnosis[All Fields] OR tele-health[All Fields] OR tele-monitoring[All Fields] OR (virtual[All Fields] AND care[All Fields]) OR "virtual clinic"[All Fields] OR (smart[All Fields] AND care[All Fields]) OR (("health"[MeSH Terms] OR "health"[All Fields]) AND app[All Fields]) OR (intelligent[All Fields] AND ("health"[MeSH Terms] OR "health"[All Fields])) OR "electronic health"[All Fields] OR ("Digit Health"[Journal] OR ("digital"[All Fields] AND "health"[All Fields]) OR "digital health"[All Fields]) OR ("videoconferencing"[MeSH Terms] OR "videoconferencing"[All Fields]) OR ("videoconferencing"[MeSH Terms] OR "videoconferencing"[All Fields] OR "videoconference"[All Fields]) OR ("remote consultation"[MeSH Terms] OR ("remote"[All Fields] AND "consultation"[All Fields]) OR "remote consultation"[All Fields]))	**122**
WoS (Topic)	"TS=(((carbon footprint) OR (footprint) OR (ecological footprint) OR (greenhouse gas) OR (""life cycle assessment"") OR co2eq OR co2e OR (carbon dioxide equivalent) OR (co2 equivalent) OR co2-eq OR (carbon emission) OR (carbon reduction)) AND (telemedicine OR teleconsultation OR telecare OR telediagnosis OR telehealth OR telemonitoring OR tele-medicine OR tele-consultation OR tele-care OR tele-diagnosis OR tele-health OR tele-monitoring OR (virtual care) OR ""virtual clinic"" OR (smart care) OR (health app) OR (intelligent health) OR ""electronic health"" OR (digital health) OR videoconferencing OR videoconference)) Indexes=SCI-EXPANDED, SSCI, A&HCI, CPCI-S, CPCI-SSH, BKCI-S, BKCI-SSH, ESCI, CCR-EXPANDED, IC Timespan=All years"	**240**
Scopus	TITLE-ABS-KEY (((carbon AND footprint) OR (footprint) OR (ecological AND footprint) OR (greenhouse AND gas) OR ("life cycle assessment") OR co2eq OR co2e OR (carbon AND dioxide AND equivalent) OR (co2 AND equivalent) OR co2-eq OR (carbon AND emission) OR (carbon AND reduction)) AND (telemedicine OR teleconsultation OR telecare OR telediagnosis OR telehealth OR telemonitoring OR tele-medicine OR tele-consultation OR tele-care OR tele-diagnosis OR tele-health OR tele-monitoring OR (virtual AND care) OR "virtual clinic" OR (smart AND care) OR (health AND app) OR (intelligent AND health) OR "electronic health" OR (digital AND health) OR videoconferencing OR videoconference OR (remote AND consultation))) AND ((LIMIT-TO (DOCTYPE , "ar") OR LIMIT-TO (DOCTYPE , "bk") OR LIMIT-TO (DOCTYPE , "re") OR LIMIT-TO (DOCTYPE , "ch") OR LIMIT-TO (DOCTYPE , "cp")) AND (LIMIT-TO (LANGUAGE , "English") OR LIMIT-TO (LANGUAGE , "German"))	**492**
CINAHL	TX (((carbon footprint) OR (footprint) OR (ecological footprint) OR (greenhouse gas) OR ("life cycle assessment") OR co2eq OR co2e OR (carbon dioxide equivalent) OR (co2 equivalent) OR co2-eq OR (carbon emission) OR (carbon reduction)) AND (telemedicine OR teleconsultation OR telecare OR telediagnosis OR telehealth OR telemonitoring OR tele-medicine OR tele-consultation OR tele-care OR tele-diagnosis OR tele-health OR tele-monitoring OR (virtual care) OR "virtual clinic" OR (smart care) OR (health app) OR (intelligent health) OR "electronic health" OR (digital health) OR videoconferencing OR videoconference OR (remote consultation)))	**38**
EconBiz	(((carbon AND footprint) OR (footprint) OR (ecological AND footprint) OR (greenhouse AND gas) OR ("life cycle assessment") OR co2eq OR co2e OR (carbon AND dioxide AND equivalent) OR (co2 AND equivalent) OR co2-eq OR (carbon AND emission) OR (carbon AND reduction)) AND ((telemedicine OR (remote AND consultation) OR teleconsultation OR telecare OR telediagnosis OR telehealth OR telemonitoring OR tele-medicine OR tele-consultation OR tele-care OR tele-diagnosis OR tele-health OR tele-monitoring OR (virtual AND care) OR "virtual clinic" OR (smart AND care) OR (health AND app) OR (intelligent AND health) OR "electronic health" OR (digital AND health) OR videoconferencing OR videoconference)))	**430**
		**1322**
