# Supplementary material for: A Transparency Checklist for Carbon Footprint Calculations Applied within a Systematic Review of Virtual Care Interventions
Source: Int J Environ Res Public Health. 2022 Jun 18;19(12):7474. doi: 10.3390/ijerph19127474 (PMC9223517; doi:10.3390/ijerph19127474)
Supplement: Supplementary file 1 [file ijerph-19-07474-s001.zip › Supplementary S2 Further description of transparency catalogue.pdf]

Supplementary S2:
Further description of transparency catalogue

Item	Assessment question	How to extract	Further description of item
			**Aim**
1	Does the study specify its aim, e.g. in terms of the product or service for which CF is assessed?	Assess whether the healthcare goods or services subject to the study are described; if yes, extract a brief description. In particular, extract the name of the product subject to the assessment; its comparator and the aim of comparison if applicable; and its predecessor and the aim of performance tracking if applicable.	At the onset of a CF calculation, the aim of the study needs to be determined and all methodological choices need to be made relative to that aim. Generally, the aim of a CF study is to quantify the potential contribution of a good (which may be a product or a service) to global warming by quantifying all significant GHG emissions and removals it involves [Ref. ISO 14067:2018, 6.3.1]. However, this may be done to pursue different, more specific goals. Three business goals mentioned by the GHG protocol are product differentiation, climate change management, and performance tracking [Ref. GHG Prot., 2]. In the first case, it is of particular importance to assess and present a single product's CF in a comprehensive and transparent manner. In the second case, the CF of different products or services may be compared. For this purpose, it needs to be ensured that scope, data, methods of analysis and presentation of results are identical or equivalent. Processes that are equal in the two products can reasonably be excluded (see [Ref. ISO 14067:2018, Annex B] and the GHG protocol for specific guidance). In the third case, if the CF of one product is tracked over time, additional requirements apply (see [Ref. ISO 14067:2018, 6.4.7] and [GHG Prot., 14] for further guidance)
			**Scope**
2a	Does the study specify the functional unit?	Assess whether a functional unit is specified; if yes, extract the functional unit	CF should be reported in relation to some unit of analysis that facilitates comparisons. In the case of a full CF, the final use of the product is known, and the unit of analysis can be defined in terms of a functional unit, i.e. the quantified performance of the product. For example, rather than reporting the annual CF of running a server to be used for a telemedicine service, it would be preferable to calculate the CF per treated patient because this can then be compared to the CF per treated patient without this service. This functional unit needs to be consistent with the aim of the CF study [Ref. ISO 14067:2018, 5.3; 6.3.3]. Following the GHG protocol, in addition to the function (service) a product fulfils, the definition of a functional unit typically includes the amount of time needed to fulfil the function and the expected quality level [Ref. GHG Prot. 6.2]. If a study aims to assess only the CF per unit of a product rather than per functional unit, then the study provides a partial CF only [Ref. ISO 14067:2018, 5.3; 6.3.3]
2b		Assess whether no final use of the product is known and/or whether the study explicitly justifies the limitation of a partial carbon footprint; if yes, report 'Partial CF'	
3	Does the study specify the reference flow?	Assess whether the reference flow is specified; if yes, extract information about the reference flow	A specific amount of inputs and outputs is needed to perform a functional unit. This amount is termed the 'reference flow' [Ref. ISO 14067:2018, 3.1.3.9]. For example, the recommended dose of a drug could be 2 grams or one diagnostic teleconsultation may require 20 minutes of server time, 5 megabytes of data transfer, etc. In conjunction with the functional unit, the amount of inputs and outputs needed to perform the functional unit needs to be specified. These amounts of inputs and outputs form the basis for calculating the CF [Ref. GHG Prot. 6.3.2]. In partial CFs where the functional unit is unknown, the unit of analysis is the reference flow [Ref GHG Prot. 6.2]
4	Does the study provide a description of the life cycle stages?	Assess whether the life cycle phases of the product or service under investigation are explicitly addressed and described; if yes, extract stated life cycle phases	GHG emissions occur during the use of a product and the total span of its life cycle, i.e. during material acquisition and re-processing; production; distribution and storage; use; and end-of-life treatment. All of these stages need to be described and included in a CF analysis to account for all relevant inputs and outputs. For some products, they may need to be adapted to better reflect the specific product's life cycle [Ref. GHG Prot. 7.2]

5	Does the study provide a list of important unit processes?	Assess whether a list of unit processes is provided; if yes, extract the list of unit processes	A CF study should include all attributable processes, i.e. all relevant service, material and energy flows that become the product, make it, and carry it through its life cycle [Ref GHG Prot. 7.2]. To facilitate comparisons between CF studies and to allow the reader to judge whether all relevant elements are included, CF studies need to provide a list of all included unit processes (i.e. a list of the smallest elements for which inputs and outputs are quantified) [Ref. ISO 14067:2018, 3.1.3; 3.1.3.6]
6	Does the study specify exclusions and reasons for exclusions?	Assess whether exclusions of unit processes or single energy or material flows are reported; if yes, extract a list of data exclusions	Even if all attributable processes ought to be included, certain flows may be found to be insignificant for the CF calculation and therefore be excluded for practical reasons. These need to be reported as data exclusions. They should be based on cut-off criteria that are explicitly defined and consistently applied [Ref. ISO 14067:2018, 6.3.4.3]
7	Does the study specify the system boundary?	Assess whether, in the methods section, the system boundary is specified and justified. If yes, extract information on the system boundary	The system boundary identifies which processes are included in the analysis [Ref. GHG Prod 7.1]. Generally, the GHG Product Standard recommends the boundary for final products to include the complete life cycle, i.e. from cradle-to-grave. For intermediate products, and if the function of the final product (for which the intermediate product is an input) is not known, the GHG Product Standard recommends a cradle-to-gate boundary. Here, GHG emissions and removals are only considered from material acquisition through to when the product leaves the company's gate [Ref. GHG Prot. 7.2]. Generally, all decisions about the system boundary need to be specified and justified, and they need to be made with reference to the aim of the CFP study [Ref. ISO 14067:2018, 6.3.4]
		**<u>Data</u>**	
8	Does the study provide sources for all data used in the analysis?	Assess whether all data sources are provided (these may include primary and secondary data); if yes, extract all data sources	CF analyses require data on the amount of all inputs and outputs and the GHG emissions associated with them. For the most important processes and for processes under own ownership or control, site-specific primary data should be collected. If no primary data of sufficient quality are available, secondary data can be used. These include, for example, data from life cycle databases like the GHG emissions associated with production and use of a specific type of server. The sources of all data in an analysis need to be specified to allow the comparison of different CF analyses and to judge the appropriateness of data inputs. The GHG Protocol Product Standard recommends using a structured process starting with a data management plan for collecting the data and assessing their quality [Ref. ISO 14067:2018, 6.3.5]; [Ref. GHG Prot., 8.2, 8.3]
9	Does the study assess the temporal representativeness of the data?	Assess whether the data year or other details about the period for which the data are relevant are reported; if yes, extract exemplary information on temporal representativeness	Besides data sources, CF analyses should also provide information about the quality of their input data. One indicator of quality is temporal representativeness: Technologies and scientific knowledge about associated GHG emissions can change. For example, a server today may be very different from a server 15 years ago in terms of the energy it consumes and its construction material. As another example, energy consumption for heating typically differs between winter and summer. Therefore, the age or timing of the data used in the analysis needs to be assessed and documented to ensure it is representative of the process for which it was collected [Ref. ISO 14067:2018, 6.3.5] [GHG Prot. 8.2]
10	Does the study assess the geographical representativeness of the data?	Assess whether information about the geographical region to which the data apply is reported; if yes, extract exemplary information on geographical representativeness	A second indicator of data quality is geographical representativeness: GHG emissions of certain activities may not be transferable across regions or countries. For example, energy use for heating an outpatient facility is likely to differ between Southern Italy and Northern Finland. Additionally, the emissions associated with electricity use vary depending on the energy mix of the electrical grid in the country or region where electricity is used. Therefore, the geographical representativeness of data needs to be assessed and documented to allow the reader to assess whether the data are appropriate for the study [Ref. ISO 14067:2018, 6.3.5 b] [GHG Prot. 8.2]

11	Does the study assess the technological representativeness of the data?	Assess whether information about the technology for which the data are relevant is reported; if yes, extract exemplary information on technology coverage	A third indicator or data quality is technological representativeness: GHG emissions for specific unit processes (e.g. travelling from home to outpatient facility) depend on the technologies (e.g. means of transport) used. Therefore, the technology coverage of the data also needs to be assessed and documented in a CFP study [Ref. ISO 14067:2018, 6.3.5 c] [GHG Prot. 8.2]
12	Does the study assess the completeness of the data?	Assess whether information about the completeness is provided; if yes, extract this information	A fourth indicator of data quality named in all three standards for assessing CF is completeness. Even if it is defined slightly differently in the standards, assessing completeness always includes the need to estimate what percentage of the total flow is measured or estimated by the data [Ref. ISO 14067:2018, 6.3.5 e]
		**Analysis**	
13	Does the study estimate CF in terms of CO_2e?	Assess whether an outcome is specified in terms of CO_2e [Ref. ISO 14067:2018, 7.2]; if yes, state 'yes'	Besides CO_2, there are other GHGs that contribute to global warming. One example relevant to healthcare is N2O, which is commonly used in anaesthesia [25]. To account for these different GHGs, studies should assess CF not in terms of CO_2 only but in terms of CO_2 equivalents (CO_2e), a unit that integrates the impact of all of these different GHGs into one single figure
14a	Does the study provide a list of GHGs taken into account?	Assess whether a list of GHGs taken into account is provided [Ref. ISO 14067:2018, 7.3 e)]; if yes, extract included GHGs	CF studies should include all GHGs relevant to the product system under analysis. A list of included GHGs should be provided [Ref. ISO 14067:2018, 7.3 e] to ensure transparency about which GHG are included in the assessment. The GHG Product Standard further specifies that generally, at least the GHGs carbon dioxide (CO_2), nitrous oxide (N2O), sulphur hexafluoride (SF6), perfluorocarbons (PCFs) and hydrofluorocarbons (HCFs) are to be included in the analysis and may be mentioned in the report [GHG 041613, 6.2]
14b		If CO_2 is analysed only, has it been justified why this is the only relevant GHG? If yes, extract the justification on which it was based (see also item 5)	
15a	Does the study specify the selected characterisation factors?	Assess whether characterisation factors are reported; if yes, extract the source of the values	The mass of other GHGs is to multiplied by a characterisation factor comparing the global warming potential of each single GHG to that of CO_2 to account for these other GHGs in the calculation of CO_2e. An authoritative source for these index values is the IPCC report. As an example, the effect of one unit mass of N2O on global warming for a duration of 100 years is 265 times as high as the effect of one unit mass of CO_2 [IPCC 2006, 1535]. Thus, 1 g emission of N2O ought to be included as 265 g CO_2e in the aggregated results. [Ref. ISO 14067:2018, 3.1.2]
15b		If CO_2 is analysed only, has it been justified why this is the only relevant GHG? If yes, extract justification it was based on (see also items 5 and 12b)	
16a	Does the study report the selected allocation procedures?	Assess whether allocation procedures are addressed. If yes, extract shared processes and allocation procedures	Typically, product life cycles contain at least one common process that has various products as inputs or outputs and for which it is impossible to collect data at the individual input or output level. For these common processes, the total emissions or removals must be partitioned among the multiple inputs or outputs, a challenging element of a CF analysis also known as allocation [Ref. GHG Product Standard 9.1]. For example, while a server's infrastructure and its energy consumption may be associated with a hospital's telehealth service, it may, at the same time, be used to host the hospital's website and to transmit data to sickness funds for reimbursement purposes. In such cases, the identification of processes shared with other product systems is needed. Unless allocation can be avoided, e.g. by distinguishing between different subsystems (e.g. servers, one of which hosts the telehealth service only), inputs and outputs should be partitioned to reflect the underlying physical relationships between the different products (e.g. computing power needed for the different applications) or other relationships like their economic value [Ref. ISO 14067:2018, 6.4.6.2]
16b		If no allocation is addressed, was it why allocation is not relevant to the study justified? If yes, extract justification (see also item 5)	

		Results	
17	Does the study report the outcomes per unit of analysis?	Assess whether data on CF per unit of analysis is provided; if yes, extract the figure and the unit of analysis	The core result of a CF analysis is the total outcome per unit of analysis. As stated above, the preferred unit of analysis is the functional unit if the final use of the product is known. The unit of analysis needs to be consistent with the aim of the CF study [Ref. ISO 14067:2018, 5.3; 6.3.3]
18	Does the study report CF separately per specific component?	Assess whether CF is reported separately per component; if yes, extract component and CFP per component (which may include that some components of emissions or removals amount to zero)	In addition to aggregated results, ISO and GHG Prot. require that CF studies should also report the results per specific component. ISO 14067:2018, 6.4.9 provides several requirements for reporting specific GHG emissions and removals where different approaches may lead to different results. GHG Prot. 11.2 also provides additional requirements. Both guidelines state that fossil and biogenic GHG emissions and removals should be included and reported separately. Due to land use change (e.g. due to changes in above- and below ground biomass over time), GHG emissions should also be reported separately when applicable [Ref. ISO 14067:2018, 6.4.9]
19	Does the study report CF according to life cycle phases?	Assess whether total GHG emissions are differentiated by life cycle phases; if yes, extract data	To ensure that all relevant GHG emissions are accounted for and to guide the search for improvements, GHG Prot., ISO and PAS2050 agree that CF needs to be reported by life cycle phases. [Ref. ISO 14067:2018, 6.3.1; GHG Prot., 11.2.; PAS2050, 9]
20	Does the study report a qualitative statement on the influence of key uncertainties or methodological choices on the result?	Assess whether the impact of at least one uncertainty or methodological assumption on results is reported; if yes, extract the most influential ones	CF results are subject to uncertainty. One important source is the uncertainty of input data (see items 8–12 above). Another important source is methodological choices, such as decisions about the method of allocation (see item 16 above). Additionally, assumptions about a product's use profile and its end-of-life may have implications for its total CF. For example, reductions in travel emissions induced by a new telehealth service may be outweighed by an increase in material or energy flow because the new service is used more frequently. Additionally, it is likely to make a difference to the CF if a product is recycled or incinerated. Therefore, CF studies should at least include a qualitative assessment of the influence of this uncertainty on the final results
		**Critical interpretation**	
21	Does the study perform a quantitative sensitivity analysis?	Assess whether quantitative sensitivity analyses are reported; if yes, extract type of sensitivity analysis (e.g. one-way or two-way sensitivity analysis, tornado diagram, probabilistic analysis)	While the GHG Prot. reporting requirements focus on qualitative assessment; quantitative analysis of uncertainty can add clarity and transparency to the reader. It can also help prioritise efforts to improve data quality in those areas of uncertainty that contribute most to the overall uncertainty of the results. As an example, if no CF for a server infrastructure needed for a telehealth service is available, data from older hardware may be used, and the impact of different values on total CF could be assessed and compared with the impact of different use scenarios. Therefore, when available, results from quantitative uncertainty analysis should be reported [Ref. ISO 14067:2018, 7.3 k; GHG Prot. 13.3.5]
22	Does the study critically discuss limitations, e.g. appropriateness of system boundary, data quality, or methods of analysis?	Assess whether limitations of the CF study are critically discussed; if yes, extract exemplary reported limitations	A CF calculation contains various assumptions and potential sources of errors, e.g. in the selection of processes with highest impact to include or in the selection and analysis of the data. In addition to calculation errors, a CF analysis may also contain value judgements that may or may not be shared by the reader. Additionally, CF calculations are always subject to the limitation that other environmental impacts (e.g. other emissions impacting on other environmental resources like water use) are omitted from the analysis. Therefore, a CF should be complemented by a critical discussion of limitations [Ref. ISO 14067:2018, 7.3m; 6.6; Annex A3]

Abbreviations: CF = Carbon Footprint, CO_2 = carbon dioxide; CO_2e = carbon dioxide equivalent; GHG = Greenhous gases; IPCC = Intergovernmental Panel on Climate Change
