# Supplementary material for: A Transparency Checklist for Carbon Footprint Calculations Applied within a Systematic Review of Virtual Care Interventions
Source: Int J Environ Res Public Health. 2022 Jun 18;19(12):7474. doi: 10.3390/ijerph19127474 (PMC9223517; doi:10.3390/ijerph19127474)
Supplement: Supplementary file 1 [file ijerph-19-07474-s001.zip › Supplementary S4_Savings.pdf]

Supplementary S4: Savings of CO_2e / CO_2 per patient or consultation

	Extraction				*Calculation			
Author	Total savings CO2e	Total savings CO2 (if CO2e not reported)	Number of patients	Number of consultations	CO2e savings per patient	CO2e savings per consultation	CO2 savings per patient	CO2 savings per consultation
Andrew et al. 2018	-51000	n.a.	45	263	-1133,33	-193,92	-	-
Connor et al 2011	-241,47	n.a.	30	60	-8,05	-4,02	-	-
Connor et al 2019	-1815	n.a.	1008	Not reported	-1,80	-	-	-
Dorrian et al. 2019	CO2e not reported	n.a.	42	42	-	-	-123,00	-
Dullet et al. 2017	CO2e not reported	-1969	11281	19246	-	-	-0,17	-0,10
Holmner et al 2014	Savings not reported	Savings n. r.	Savings n. r.	719	-	-	-	-
Lewis et al 2009*	CO2e not reported	-2950	90	30	-	-	-32,78	-98,33
Masino et al. 2010	-185159	n.a.	Not reported	840	-	-220,43	-	-
Miah et al 2018	-900	n.a.	409	Not reported	-2,20	-	-	-
Oliveira et al. 2013	-455000	n.a.	-	20824	-22,00	-21,85	-	-
Paquette et al. 2019	CO2e not reported	-1632	87	146	-	-	-18,76	-11,18
Smith et al 2013	Savings not reported	Savings n. r.	1000	Not reported	-	-	-	-
Turley et al 2011*	CO2e not reported	n.a.	Not reported	Not reported	-	-	-	-
Vidal-Alaball et al 2019	CO2e not reported	-29384	Not reported	9034	-	-	-	-3,25
Whetten et al. 2019	-618772	n.a.	Not reported	2020	-306,00	-306,32	-	-
Wiljer et al. 2012	-6945,6	n.a.	113	Not reported	-61,47	-	-	-
Blenkinsop et al 2021	-37500	n.a.	1277	1667	-29,37	-22,50	-	-
Bonsall 2020	-680	n.a.	61	Not reported	-11,15	-	-	-
Filfilan et al 2021	-1141	n.a.	80	Not reported	-14,26	-	-	-
O Conell 2021	CO2e not reported	-15370	Not reported	1476	-	-	-	-10,41
Schulz et al 2021	CO2e not reported	-22370	58	Not reported	-	-	-385,69	-
Sellars et al. 2020	-2113	n.a.	50	Not reported	-42,26	-	-	-
Jiang et al 2021	CO2e not reported	-35500	366	560	-	-	-96,99	-63,39
				**Count**	11	6	6	6
				***Average**	-148,35	-128,17	-109,57	-31,11
				**Std Dev**	322,39	117,23	130,83	36,80
				**Min**	-1133,33	-306,32	-385,69	-98,33
				**Max**	-1,80	-4,02	-0,17	-0,10

Underlined, if the value was extracted directly
*Lewis et al.: Only second survey
*Turley et al.: Ecological footprint
*Average: Sum of available values divided by number of all available values of the column
*Calculation: Total savings CO2e or CO2 divided by number of patients or consultations
Abbreviation: n. r. = Not reported
