# Supplementary material for: A Transparency Checklist for Carbon Footprint Calculations Applied within a Systematic Review of Virtual Care Interventions
Source: Int J Environ Res Public Health. 2022 Jun 18;19(12):7474. doi: 10.3390/ijerph19127474 (PMC9223517; doi:10.3390/ijerph19127474)
Supplement: Supplementary file 1 [file Supplementary S3_Extracted results.pdf › Supplementary S3_Extracted results.pdf]

Please use this table as electronic supplementary material by using the zoom function (CTRL+Scroll).

Item	Assessment question	Andrew et al. 2018	Connor et al 2011	Connor et al 2019	Dorrian et al. 2019	Dullet et al. 2017	Holmner et al 2014	Lewis et al 2009
1	Does the study specify its aim, e.g. in terms of the product for which CFP is assessed?	Describe a telehealth model of care for routine follow up of renal transplant recipients in a tertiary centre	Report the use of telephone consultation clinics to provide follow-up to renal transplant recipients	Evaluate the clinical, fiscal and environmental impact of a specialist-led acute ureteric colic virtual clinic pathway	Assess feasibility and carbon emission reductions of ENT tele-endoscopy for head and neck cancer assessment	Estimate travel-related and environmental savings resulting from the use of telemedicine for outpatient specialty consultations with a university telemedicine program	Assess the potential of telerehabilitation services based on videoconferencing to reduce travelling at the University Hospital of Northern Sweden and thus carbon emissions in healthcare	Evaluate the environmental impact of using video conferencing versus attending a meeting in person
	Categorization for figure 3	*Tertiary care*	*Tertiary care*	*Secondary care*	*Secondary care*	*Secondary care*	*Tertiary care*	*Tertiary care*
2	Does the study specify the functional unit?	Not reported	Not reported	Not reported	Patient	Consultation	Consultation	Not reported
	Categorization for figure 3	Not reported	Not reported	Not reported	Patient	Consultation	Consultation	Not reported
3	Does the study specify the reference flow?	(study only considered total of 203,202 km in travel distance avoided)	(study only considered 39,4 km of travel on average)	(study only considered mean distance travelled of 4.3 miles per patient)	(study only considered 42km of road travel and 303 km of flight distance one way avoided)	(Study only considered mean per-consultation round-trip distance savings of 278 miles)	One hour of meeting duration	Not reported
	Categorization for figure 3	*(only travel distance avoided)*	*(only travel distance avoided)*	*(only travel distance avoided)*	*(only travel distance avoided)*	*(only travel distance avoided)*	*1 hour of meeting duration*	*Not reported*
4	Does the study provide a description of the life cycle stages?	Not reported	Not reported	Not reported	Not reported	Not reported	(Life cycle emissions included but taken from secondary data sources only)	Not reported
5	Does the study provide a list of important unit processes?	Not reported	Not reported	Not reported	Not reported	Not reported	Life cycle inventories on hardware and software required to connect patient and specialist; life cycle carbon emissions of travelling	Not reported
6	Does the study specify exclusions and reasons for exclusion?	Not reported	Not reported	Not reported	Not reported	Not reported	Not reported	Not reported
7	Does the study specify the system boundary?	Not reported	Not reported	Not reported	Not reported	Not reported	Addressed indirectly by referring to a paper by Ong et al. [Ref noch einfügen]	Not reported
8	Does the study provide sources for all data used in the analysis?	Distances between patients' postcode and hospital obtained from Geo-Coded Natioonal Address File; CO_2 emission based on mean calculation (251g CO_2 emitted per km)	Primary data on patients regarding their means of transport used; distances based on postcode and google maps	Primary data on patients regarding their means of transport used; distances based on postcode and google maps	Few secondary data (distance between hospitals, travel emissions)	Patient addresses from telemedicine consultation database at the University of California Davis Health System; distance calculation from MP Mileage 2.5 Software; pollutant values from Evironmental Protection Agency Office of Transportation and Air Quality	Data from 238 telemedicine appointments in hand- and plastic surgery section and 481 therapy sessions in speech therapy unit to assess travel distances and use of devices; expert estimates for duration of appointments; published sources for life cycle carbon emissions associated with devices and travelling	Questionnaire data regarding distance travelled, vehicle engine size, whether petrol or diesel, number of passengers; CO_2 savings estimated using the UK government's calculator
	Categorization for figure 3	*Institutional and public secondary data*	*Different primary and secondary data*	*Different primary and secondary data*	*Public secondary data*	*Institutional and public secondary data*	*Different primary and secondary data*	*Different primary and secondary data*
9	Does the study assess the temporal representativeness of the data?	Not reported	Not reported	Not reported	Not reported	Not reported	Not reported	Not reported
10	Does the study assess the geographical representativeness of the data?	(Location-specific travel, country specific gasoline consumption of average cars)	(Location-specific travel, country specific gasoline consumption of average cars)	(Location-specific travel, country specific gasoline consumption of average cars)	(Location-specific travel, country specific gasoline consumption of average cars)	(Location-specific travel, country specific gasoline consumption of average cars)	Rural region in Sweden for travel distances and use of devices	(Location-specific travel, country specific gasoline consumption of average cars)
	Categorization for figure 3	*Average travel emissions only*	*Average travel emissions only*	*Average travel emissions only*	*Average travel emissions only*	*Average travel emissions only*	*Yes, based on region*	*Average travel emissions only*
11	Does the study assess the technological representativeness of the data?	Not reported	2009 guidelines to DEFRA / DECC's GHG conversion factors for company reporting for specific means of transport (car, public transport)	Not reported	Not reported	Not reported	Vehicules for travel: most commonly purchased petrol and diesel cars in Europe in 2010; details about technologies following a published study [Ref. Ong et al.]	Not reported
	Categorization for figure 3	*Not reported*	*Some information based on published averages*	*Not reported*	*Not reported*	*Not reported*	*Some information based on published averages*	*Not reported*
12	Does the study assess the completeness of the data?	Not reported	Not reported	Not reported	Not reported	Not reported	Not reported	Not reported
13	Does the study estimate CFP in terms of CO_2e?	Yes	Yes	Yes	Not reported	Not reported	Yes	Not reported
	Categorization for figure 3	*Yes*	*Yes*	*Yes*	*Not reported*	*Not reported*	*Yes*	*Not reported*
14a	Does the study provide a list of GHGs taken into account?	Not reported	Not reported	Not reported	Not reported	Carbon dioxide, Carbon monoxide, Nitrogen oxides, Volatile organic compounds	Not reported	Not reported
	Categorization for figure 3	Not reported	Not reported	Not reported	Not reported	Yes	Not reported	Not reported
15a	Does the study specify the selected characterization factors?	Not reported	DEFRA conversion factors	Not reported	Not reported	Not reported	Not reported	Not reported
	Categorization for figure 3	*Not reported*	*Based on national standard*	*Not reported*	*Not reported*	*Not reported*	*Not reported*	*Not reported*
16a	Does the study report the selected allocation procedures?	Not reported	Not reported	Not reported	Not reported	Not reported	Processes appear to be segregated to disjunct subprocesses: carbon emissions were estimated per unit of time devices are used for all devices or per unit of distance travelled; therefore, allocation appears not to be relevant.	Not reported
	Categorization for figure 3	*Not reported*	*Not reported*	*Not reported*	*Not reported*	*Not reported*	*Some information provided*	*Not reported*
17	Does the study report the outcomes per unit of analysis?	(51 metric tons of CO_2eq saved during 263 clinical reviews for 45 patients; outcomes per unit of analysis not reported)	(Reduction of 2,818 kg CO_2eq for annual load of 350 telephone consultations; outcomes per unit of analysis not reported)	(Total savings of 0.70–2.93 metric tonnes of carbon dioxide equivalent for 1008 patients; outcomes per unit of analysis not reported)	123 kg CO_2 avoided per person	(Among 11,281 patients, the following savings were achieved: carbon dioxide 1,969 metric tons; Carbon monoxide 50 metric tons, Nitrogen oxides 3.7 metric tons, Volatile organic compounds 5.5 metric tons; outcomes per unit of analysis not reported)	1.86-8.43 kgCO_2e per hour of telemedicine appointment, without avoided travel	(1696 kg of CO_2 emission avoided in first period with over 60 patients, 2590 kg of CO_2 emission in second period with over 90 patients; outcomes per unit of analysis not reported)
	Categorization for figure 3	*Not reported*	*Not reported*	*Not reported*	*Carbon savings*	*Not reported*	*Carbon savings*	*Not reported*
18	Does the study report CFP separately per specific component?	Not reported	Not reported	Not reported	Not reported	Not reported	Not reported	Not reported
	Categorization for figure 3	*Not reported*	*Not reported*	*Not reported*	*Not reported*	*Not reported*	*Not reported*	*Not reported*
19	Does the study report CFP according to life cycle phases?	Not reported	Not reported	Not reported	Not reported	Not reported	Not reported	Not reported
20	Does the study report a qualitative statement on the influence of key uncertainties or methodological choices on the result?	Not reported	Not reported	In a rural setting it is it is likely the environmental impact would be threefold greater	Not reported	Not reported	Sensitivity analysis e.g. regarding different use rates of the equipment (ten-time reduction in use rate of equipment would increase emission by 20% for one hour meeting) or different cars for traveling	Not reported
	Categorization for figure 3	*Not reported*	*Not reported*	*≤ 2 variations considered*	*Not reported*	*Not reported*	*> 2 variations considered*	*Not reported*
21	Does the study perform a quantitative sensitivity analysis?	Not reported	Not reported	Not reported	Not reported	Assumption that only 90%, 75% or 50% of telemedicine visits avoid travel to healthcare facility - with respective decreases in CFP	Sensitivity analysis e.g. regarding 5% reduction of all life cycle phases of a private vehicule, different types of hardware, different bandwiths, etc.	Not reported
	Categorization for figure 3	*Not reported*	*Not reported*	*Not reported*	*Not reported*	*≤ 2 variations considered*	*> 2 variations considered*	*Not reported*
22	Does the study critically discuss limitations, e.g. appropritateness of system boundary, data quality, or methods of analysis?	limitations in patient feedback and outcome data, general limitations of telehealth	Not reported	Study ignored that some patients may have cycled or walked to facility	Not reported	Travel emissions may differ from US average values	Uncertainty about future trends in automotive and digital technologies; also about the use of telemedicine on usual care	Not reported
	**Transparency score**	**0.18**	**0.23**	**0.23**	**0.18**	**0.27**	**0.64**	**0.14**

Item	Assessment question	Masino et al. 2010	Miah et al 2018	Oliveira et al. 2013	Paquette et al. 2019	Smith et al 2013	Turley et al 2011	Vidal-Alaball et al 2019	Whetten et al. 2019
1	Does the study specify its aim, e.g. in terms of the product for which CFP is assessed?	Estimate the reduction in GHG emissions resulting from telemedicine consultations	Quantify the clinical, financial and environmental benefits of virtual urology clinic	Compare the emissions of patients attending a teleconsultation – an outpatient appointment using video-conferencing equipment – with those of patients attending a face-to-face appointment	Analyze the impact of outpatient telemedicine services versus hypothetical in-person traditional consultations on the travel burden of vascular surgery patients with regard to the emission of environmental pollutants	To estimate the carbon footprint of four behavioural support services for smoking cessation - text message support, telephone counselling, group counselling and individual counselling I: text message support	Assess the Eco-Health Footprint of Kaiser Permanente's electronic health record system KP HealthConnect™	Assess the effect of a telemedicine program developed in the central Catalan region in lowering the environmental footprint by reducing the emission of atmospheric pollutants	Assess GHG emissions resulting from neuro-emergent telemedicine consultations to estimate GHG reductions associated with nationwide roll-out
	Categorization for figure 3	*Quarternary care*	*Secondary care*	*Secondary care*	*Tertiary care*	*Prevention*	*Support system*	*Secondary care*	*Tertiary care*
2	Does the study specify the functional unit?	Not reported	Not reported	Patient	Not reported	Patient; lifetime quitter	Not reported	(Journey or patient)	Patient
	Categorization for figure 3	Not reported	Not reported	Patient	Not reported	Final health outcome	Not reported	Patient	Patient
3	Does the study specify the reference flow?	Mean of 473 km of avoided travel and energy consumption of 1 hour of videoconferencing unit use per consultation included	(Study only considered 4,623 travel miles avoided)	Study only included average of 5.9 km travel each way to for telemedicine appointments compared to average of 47 km travel of face-to-face patients	The study only considered 15.6 miles of average one-way distance saved by the utilization of telemedicine services	Different amounts of patient and staff travel, clinic and office space, and technology, e.g. Up to 260 messages delivered over 33 weeks; 30 minutes of data monitoring per participant	Specific amounts of paper use: e.g. health records, X-ray jackets, Electronic forms, Visit summaries, Personal computer packaging; Energy use: Gasoline used for transportation, Personal computers, Data centers; Waste: X-ray plastics, Personal computer plastics	(Study only considered 21.3 km average travel distance avoided per journey saved)	Energy consumption associated with 338 miles of traveling (including helicopter transfer) avoided and 1h of computer use per patient
	Categorization for figure 3	*Meeting duration, computer use*	*(only travel distance avoided)*	*(only travel distance avoided)*	*(only travel distance avoided)*	*Detailled specification of reference flows*	*Detailled specification of reference flows*	*(only travel distance avoided)*	*Meeting duration, computer use*
4	Does the study provide a description of the life cycle stages?	Not reported	Not reported	Not reported	Not reported	(Only information about length of hardware used)	Not reported	Not reported	Not reported
5	Does the study provide a list of important unit processes?	Not reported	Not reported	Not reported	Not reported	Further details provided in online appendix, e.g. air conditioned office 6 m^2 administration office; additionally, data from secondary sources	Not reported	Not reported	Not reported
6	Does the study specify exclusions and reasons for exclusion?	Not reported	Not reported	Not reported	Not reported	Water usage and clinical waste, medication to promote smoking stopping	Minor impacts, for example, effect of visit reductions on incidental paper use in ambulatory care, such as lab slips, examination table paper rolls, and parking slips; indirect impacts, for example, the reduced use of copiers to duplicate patient records; hardware because even if it would increase waste measures, they have longer life spans than the three-year lease life of desktop computers	Public transport because it is not always easily available in rural areas; emissions that deviate from the average	Telemedicine equipment use by local physicians
7	Does the study specify the system boundary?	Not reported	Not reported	Not reported	Not reported	Not reported	Not reported	Not reported	Not reported
8	Does the study provide sources for all data used in the analysis?	Primary data on residents' postal codes and addresses of care facilities; sample electricity demand (i.e., watt) values for devices using Watts Up Pro meter; secondary data for vehicule emissions (Environment Canada's National Inventory Report: Greenhouse Gas sources and sinks in Canada, 1990-2006)	Patient records for distances to healthcare facility; data on engine capacity and type of fuel from Department for Transport	Primary information on distances travelled and modes of transport used from survey of 100 teleconsultation patients and 100 face-to-face outpatients who had appointments in the second half of 2011; secondary data on diesel and petrol use	Environmental Protection Agency's average passenger vehicle emissions per mile; patient addresses from medical records	Different published sources, e.g. NHS Sustainable Development Unit, Peters GP. Carbon footprints and embodied carbon at multiple scales. Curr Opin Environ Sustainability 2010;2:245–50	Data on inpatient and outpatient general x-ray and mammography use from regional operational reports; travel distances from member addresses and primary care building locations; prescription order rates from internal pharmaceutical distribution center; paper use from paper-purchasing records	Emission of pollutants per km from http://mobilitat.gencat.cat/ca/detalls/Article/mobilitat_emissions; distances between primary care centers and referral centers from Google maps	CO_2e emission coefficients from the Environmental Protection Agency (EPA), Energy Information Administration, and locality-specific emission conversion factors; Medicare Provider Utilization and Payment Data: Inpatient, from CMS, e.g. for number of visits without telemedicine service; energy grid emission data for New Mexico from the state energy data system
	Categorization for figure 3	*Different primary and secondary data*	*Institutional and public secondary data*	*Different primary and secondary data*	*Institutional and public secondary data*	*Public secondary data*	*Different primary and secondary data*	*Public secondary data*	*Different primary and secondary data*
9	Does the study assess the temporal representativeness of the data?	Not reported	Not reported	Not reported	Not reported	Not reported	Not reported	Not reported	Not reported
10	Does the study assess the geographical representativeness of the data?	(Location-specific travel, country specific gasoline consumption of average cars)	(Location-specific travel, country specific gasoline consumption of average cars)	(Location-specific travel, country specific gasoline consumption of average cars; due to lack of data in vehicle use in Portugal, data on vehicle sales was used)	(Location-specific travel, country specific gasoline consumption of average cars)	Representative for the English NHS	Representative for Kaiser Permanente's facilities	(Location-specific travel, country specific gasoline consumption of average cars)	Location-specific travel, country specific gasoline consumption of average cars, electrical grid emission factors for New Mexico
	Categorization for figure 3	*Average travel emissions only*	*Average travel emissions only*	*Average travel emissions only*	*Average travel emissions only*	*Yes, based on healthcare payer*	*Yes, based on healthcare payer*	*Average travel emissions only*	*Yes, based on region*
11	Does the study assess the technological representativeness of the data?	Vehicule emissions for Ontario, CA; 32" LCD videoconferencing unit with a measured electricity demand of 138 watts	Not reported	Not reported	Not reported	CFP per specific input reported, e.g. for text & phone equipment or computers	Details about resource use depending on clinical activity like X-ray based on patient records	Not reported	Specific CO_2e emission for assumed Bell 429™helicopter; telemedicine equipment and server farms
	Categorization for figure 3	*Some technology-specific detail*	*Not reported*	*Not reported*	*Not reported*	*Technology-specific details*	*Technology-specific details*	*Not reported*	*Technology-specific details*
12	Does the study assess the completeness of the data?	Not reported	Not reported	Not reported	Not reported	Not reported	Not reported	Not reported	Not reported
13	Does the study estimate CFP in terms of CO_2e?	Yes	Yes	Yes	Not reported	Yes	CO_2 sequestered by (9,200)-3,600 acres of forest	Not reported	Yes
	Categorization for figure 3	*Yes*	*Yes*	*Yes*	*Not reported*	*Yes*	*Area-based carbon footprint*	*Not reported*	*Yes*
14a	Does the study provide a list of GHGs taken into account?	CH_4, N20	Not reported	Not reported	carbon dioxide, carbon monoxide, nitric oxides, and volatile organic compounds	Not reported	Not reported	CO_2, CO, NO_X, SO_2 (claim to assess environmental rather than carbon footprint)	CO_2, CH_4, N_2O and air contaminants
	Categorization for figure 3	Yes	Not reported	Not reported	Yes	Not reported	Not reported	Yes	Yes
15a	Does the study specify the selected characterization factors?	Environment Canada GreenhouseGas Division. Canada's Greenhouse Gas Inventory 1990-2000	Not reported	Defra/DECC's GHG Conversion Factors for Company Reporting	Not reported	Not reported	US Environmental Protection Agency's greenhouse gas equivalencies calculator	Not reported	Emission factors from Environment Canada Greenhouse Gas Division, Canada's Greenhouse GasInventory 1990-2000 and multiplier for air travel (only for helicopter travel)
	Categorization for figure 3	*Based on national standard*	*Not reported*	*Based on national standard*	*Not reported*	*Not reported*	*Based on national standard*	*Not reported*	*Based on national standard*
16a	Does the study report the selected allocation procedures?	Not reported	Not reported	Not reported	Not reported	Not reported	Assumptions: 90 percent of personal computers' energy use is attributable to care delivery using the electronic health records; 50 percent of the energy use at the centers is related to KP HealthConnect	Not reported	Not reported
	Categorization for figure 3	*Not reported*	*Not reported*	*Not reported*	*Not reported*	*Not reported*	*Some information provided*	*Not reported*	*Not reported*
17	Does the study report the outcomes per unit of analysis?	Not reported	(0.35–1.45 metric tonnes of CO_2e for 409 patients, predicted 12-month avoided carbon footprint is 1.05–4.35 metric tonnes of CO_2e; outcomes per unit of analysis not reported)	22 kg CO_2e avoided per patient	(Reductions of carbon dioxide, carbon monoxide, nitric oxides, and volatile organic compounds were 1632 kg, 42,867 g, 3160 g, and 4715 g, respectively for 146 telemedicine encounters among 87 unique patients; outcomes per unit of analysis not reported)	Emissions per intervention lifetime quitter were 636 (95% CI 455 to 958) kg CO_2e for text message support, 1051 (95% CI 560 to 2873) kg CO_2e for telephone counselling, 1143 (95% CI 695 to 2270) kg CO_2e for group counselling and 2823 (95% CI 1688 to 6549) kg CO_2e for individual counselling	Not reported	3248.3 g of CO_2 avoided per avoided referral to specialist care (assumably due to telemedicine service)	0.306 metric tons of CO_2e avoided per patient
	Categorization for figure 3	*Not reported*	*Not reported*	*Carbon savings*	*Not reported*	*Positive CF*	*Not reported*	*Carbon savings*	*Carbon savings*
18	Does the study report CFP separately per specific component?	Avoidance of 185 metric tons of CO_2e in vehicle emissions; emission due to energy consumption by videoconference units 42 kg of CO_2e for 840 teleconsultations	Not reported	Not reported	Not reported	Table 2 provides details about CFP for enrolment, counseling, staff, office & counseling space, IT equipment	Paper use: Health records (4,200), X-ray jackets (240), Electronic forms (17), Visit summaries 810, Personal computer packaging 283; Energy use: Gasoline used for transportation (6,400–19,200), Personal computers 3,800, Data centers 9,500; Waste: X-ray plastics (40), Personal computer plastics 78	Not reported	0.306 metric tons of CO_2e per patient due to avoided patient transfers; 32kg of additional CO_2 due to computer use
	Categorization for figure 3	*≤ 2 components*	*Not reported*	*Not reported*	*Not reported*	*Detailled specification*	*Detailled specification*	*Not reported*	*≤ 2 components*
19	Does the study report CFP according to life cycle phases?	Not reported	Not reported	Not reported	Not reported	Not reported	Not reported	Not reported	Not reported
20	Does the study report a qualitative statement on the influence of key uncertainties or methodological choices on the result?	Not reported	Not reported	Not reported	Not reported	Increased CO_2 emissions due to longer survival of ex-smokes excluded would increase overall burden; stop smoking medication excluded which would increase smoking cessation and thus total savings per life time quitter; increasing efficiency of communication devices improves overall CFP	Switching from desktop personal computers to laptops with external monitors would reduce this impact by 2.300 acres	Not reported	Not reported
	Categorization for figure 3	*Not reported*	*Not reported*	*Not reported*	*Not reported*	*> 2 variations considered*	*≤ 2 variations considered*	*Not reported*	*Not reported*
21	Does the study perform a quantitative sensitivity analysis?	Not reported	Not reported	The incorporation of modes of transport and car engine size and fuel in the analysis led to emission estimates which were 12% smaller than those assuming all patients used an average car.	Not reported	Exclusion of CFP for staff commute	(Only the impact of different assumptions of traffic reduction on total CFP)	Not reported	Not reported
	Categorization for figure 3	*Not reported*	*Not reported*	*≤ 2 variations considered*	*Not reported*	*≤ 2 variations considered*	*≤ 2 variations considered*	*Not reported*	*Not reported*
22	Does the study critically discuss limitations, e.g. appropritateness of system boundary, data quality, or methods of analysis?	Not reported	Study ignored that some patients may have cycled or walked to facility	Not reported	E.g. limited generalizability because study based on patients of a single vascular surgeon in metropolitan area	Study could not account for uncertainty in carbon emissions because carbon emission data are available as point estimates without CIs	Model limited to major impact on patient record	Few limitations mentioned, e.g. inclusion of private vehicle use only, the exclusion of certain costs like parking or costs of telemedicine equipment	Statement that wide assumptions were made, in particular that for estimating program expansion, similar patients across hospital sites were assumed
	**Transparency score**	**0.36**	**0.18**	**0.36**	**0.27**	**0.64**	**0.55**	**0.27**	**0.59**

Item	Assessment question	Wiljer et al. 2012	Blenkinsop et al 2021	Bonsall 2020	Filfilan et al 2021	O Conell 2021	Schulz et al 2021	Sellars et al. 2020	Jiang et al 2021
1	Does the study specify its aim, e.g. in terms of the product for which CFP is assessed?	Compare email and videoconferencing to face-t--face follow-up visits for patients who had previously been treated for thyroid cancer	Calculate marginal carbon emission savings from enforced telemedicine	Calculate emission savings of a asynchronous teledermatology clinic compared to one face-to-face meeting per week; extrapolate this to estimate potential savings of all 650 dermatologists	Assess the reduction of CF related to the replacement of in-person consultations with teleconsultations	Assess environmental benefits of an enforced transition to teledermatology	(The reduction of carbon output of telehealth by avoiding travel is only the secondary outcome)	Assessed the environmental impact of a virtual care clinic for new colorectal referals in NHS highland	(estimate environmental impacts of teleoncology)
	Categorization for figure 3	*Tertiary Care*	*Tertiary Care*	*Secondary care*	*Secondary care*	*Secondary care*	*Tertiary Care*	*Secondary care*	*Tertiary Care*
2	Does the study specify the functional unit?	(Journey or patient)	Not reported	Not reported	Not reported	Not reported	Not reported	Not reported	Not reported
	Categorization for figure 3	Patient	Not reported	Not reported	Not reported	Not reported	Not reported	Not reported	Not reported
3	Does the study specify the reference flow?	(Study only mentions average estimated travel distance including return trip with 120km could be avoided)	(69,5 km median retounred journey distance); one hour of teleconusultation per week	(study only considered anaverage round trip of 55.8 km)	Mean of 83,7 km of avoided travel and energy consumption of 20 min of teleconsultation included	1476 teledermatology appointments (37,8 km of travel on average)	(Study only considered 634 km travel avoided for each telehealth consultation)	50 patients	(study only considered 154.4 miles of travel on average) or (366 Patients)
	Categorization for figure 3	*(only travel distance avoided)*	*Detailled specification of reference flows*	*(only travel distance avoided)*	*Meeting duration, computer use*	*(only travel distance avoided)*	*(only travel distance avoided)*	*(only travel distance avoided)*	*(only travel distance avoided)*
4	Does the study provide a description of the life cycle stages?	Not reported	Not reported	Not reported	Not reported	Not reported	Not reported	Not reported	Not reported
5	Does the study provide a list of important unit processes?	Not reported	Travel (fuel consumption); teleclinic (energy consumption of devices and data transmission)	Not reported	Not reported	(Study only considered travel costs)	Not reported (Only patients travel considered)	Not reported (Only patient travel)	Not reported (Study only considered patient travel)
6	Does the study specify exclusions and reasons for exclusion?	Not reported	Journeys with air or ferry travel were excluded	Not reported	Not reported	Not reported	Not reported	Follow-up patient, noncolectoral referrals were excluded	Not reported
7	Does the study specify the system boundary?	Not reported	Travel (Not reported); teleclinic: (end-use device, access network, ambient temperature conditions)	Not reported	Not reported	Not reported	Not reported	Not reported	Not reported
8	Does the study provide sources for all data used in the analysis?	Only distances traveled via Google Maps (and statement that GHG emissions were based on the methodology described by Masino et al. 2010)	Travel [Distances (ArcGIS Online11 and Gogle maps routing tools); health records for the last recorded home address; Conversion Factors from the UK Department for Business, Energy and Industrial Strategy (BEIS)];<br>Teleclinic [average energy consumption through data transmission; estimation of decreasing this consumption over the last years, ZOOM data transmission; conversion factos from UK Department for Business, Energy and Indstrial Strategy]	Primary data on a sample of patients regarding their means of transport used; distances based on postcode; data extrapolated to all possible patients	(Only travel-distances and conversion factors, but no source for energy use of teleconference equipment)	Distances google maps, emission by Irish Environmental Protection Agency	Yes, Charting transport for travel emissions in Australia	AA mileage calculator, Map My Emission and International Civil Aviation Organization for the emission calculation	distances googlemaps, source for CO_2 emission per mile (epa.gov)
	Categorization for figure 3	*Different primary and secondary data*	*Different primary and secondary data*	*Different primary and secondary data*	*Public secondary data*	*Public secondary data*	*Public secondary data*	*Public secondary data*	*Public secondary data*
9	Does the study assess the temporal representativeness of the data?	Not reported	Not reported	Not reported	Not reported	Not reported	Not reported	Not reported	Not reported
10	Does the study assess the geographical representativeness of the data?	Not reported	Travel distances are representative for Chalfont Buckinghamshire site of the ULCH National Hospital for Neurology and Neurosurgery; they use UK conversion factors	(Location-specific travel)	(Location-specific travel, country specific vehicle type)	Cork/Kerry - Urban/Rural ratio 1.32:1	Australia	NHS Highland (Scottland)	Michigan, USA
	Categorization for figure 3	*unclear*	*Yes, based on region*	*Yes, based on region*	*Average travel emissions only*	*Yes, based on region*	*Average travel emissions only*	*Average travel emissions only*	*Average travel emissions only*
11	Does the study assess the technological representativeness of the data?	Not reported	Travel: Different sized cars; Teleclinic: different data rates (video definition)	Not reported	(Travel: Different type of transport (car, public transport, feet); Technological representativeness of teleconsultation not reported)	Average car	Australian average for passenger cars	petrol car with medium-sized engine	Not reported
	Categorization for figure 3	*Not reported*	*Technology-specific details*	*Not reported*	*Technology-specific details*	*Some information based on published averages*	*Some information based on published averages*	*Some information based on published averages*	*Not reported*
12	Does the study assess the completeness of the data?	Not reported	Not reported	Not reported	Not reported	Not reported	Not reported	Not reported	Not reported
13	Does the study estimate CFP in terms of CO_2e?	Yes	Yes	Yes	Yes	Not reported	Not reported	Yes	Not reported
	Categorization for figure 3	*Yes*	*Yes*	*Yes*	*Yes*	*Not reported*	*Not reported*	*Yes*	*Not reported*
14a	Does the study provide a list of GHGs taken into account?	Not reported	CO_2, CH_4 and N_2O	Not reported	Not reported (Study only considered Methane, nitrous oxide and "other air contaminants")	Not reported	Not reported	Not reported	Not reported
	Categorization for figure 3	Not reported	Yes	Not reported	Yes	Not reported	Not reported	Not reported	Not reported
15a	Does the study specify the selected characterization factors?	Not reported	Not reported	Not reported	Not reported	Not reported	Not reported	Not reported	Not reported
	Categorization for figure 3	*Not reported*	*Not reported*	*Not reported*	*Not reported*	*Not reported*	*Not reported*	*Not reported*	*Not reported*
16a	Does the study report the selected allocation procedures?	Not reported	Not reported	Not reported	Not reported	Not reported	Not reported	Not reported	Not reported
	Categorization for figure 3	*Not reported*	*Not reported*	*Not reported*	*Not reported*	*Not reported*	*Not reported*	*Not reported*	*Not reported*
17	Does the study report the outcomes per unit of analysis?	Not reported	Not reported	Average of 11.1 kg CO_2e avoided per Patient	Not reported	(15.37 metric tonnes of CO_2 for 1476 appointements; outcomes per unit of analysis not reported)	Not reported	Virtual clinic avoids 93.2 lb CO_2e per person	(35.5 metric tons of CO_2 saved during 560 teleoncology enconters)
	Categorization for figure 3	*Not reported*	*Not reported*	*Carbon savings*	*Not reported*	*Not reported*	*Not reported*	*Carbon savings*	*Carbon savings*
18	Does the study report CFP separately per specific component?	Not reported	Travel (e.g. Average car(Proportional): 380995 kg CO_2e; Small car (proportional): 31880 kg CO_2e; Large car (Proportional): 54995 kg CO_2e; also separate for diesel & petroleum); Teleclinic (e.g. desktop pc power consumption 150w/80.6 Co_2e; 1h two-way HD video conference 0.0918kWh / 0.0214 Kg CO_2e)	Not reported	Yes, 1.1kg CO_2e for electricity consumption and 1142.3 kg co_2e for transport in the 1 month period	Not reported	Not reported	Not reported	Not reported
	Categorization for figure 3	*Not reported*	*Detailled specification*	*Not reported*	*≤ 2 components*	*Not reported*	*Not reported*	*Not reported*	*Not reported*
19	Does the study report CFP according to life cycle phases?	Not reported	Not reported	Not reported	Not reported	Not reported	Not reported	Not reported	Not reported
20	Does the study report a qualitative statement on the influence of key uncertainties or methodological choices on the result?	Not reported	Travel: (petrol vs. Diesel; longer distances calculated by google maps;); Teleclinic: (Different video definition; computer vs. Laptop vs. mobile connectivity)	Not reported	Not reported	Not reported	Not reported	Not reported	Not reported
	Categorization for figure 3	*Not reported*	*> 2 variations considered*	*Not reported*	*Not reported*	*Not reported*	*Not reported*	*Not reported*	*Not reported*
21	Does the study perform a quantitative sensitivity analysis?	Not reported	Not reported	Not reported	Not reported	Not reported	Not reported	Not reported	Not reported
	Categorization for figure 3	*Not reported*	*Not reported*	*Not reported*	*Not reported*	*Not reported*	*Not reported*	*Not reported*	*Not reported*
22	Does the study critically discuss limitations, e.g. appropritateness of system boundary, data quality, or methods of analysis?	Not reported	Probably underestimate carbon emission savings from travel, climate change only as one of multiple environmental impacts	Not reported	they did not consider the type of connection, GHG emission of car parking and roads construction, possibilty of using smartphones, additional inperson meetings before teleconsultation starts; study periode was short; small patient group;	travel by car, imprecise adresses, failure to capture nonattendance rates	Not reported	Not reported	Not reported
	**Transparency score**	**0.14**	**0.55**	**0.18**	**0.23**	**0.27**	**0.14**	**0.36**	**0.09**
